# Editorial for the Special Issue “Microorganisms in Silage”

**DOI:** 10.3390/microorganisms14020300

**Published:** 2026-01-27

**Authors:** Siran Wang, Musen Wang, Marcia De Oliveira Franco, Qing Zhang, Chunsheng Bai

**Affiliations:** 1 Institute of Animal Science, Jiangsu Academy of Agricultural Sciences, Nanjing 210014, China; 2 Key Laboratory for Crop and Animal Integrated Farming, Ministry of Agriculture and Rural Affairs, Nanjing 210014, China; 3 School of Tropical Agriculture and Forestry, Hainan University, Danzhou 571737, China; wangms@hainanu.edu.cn; 4 Production Systems, Natural Resources Institute Finland (Luke), FI-31600 Jokioinen, Finland; marcia.franco@luke.fi; 5 College of Grassland Science, Qingdao Agricultural University, Qingdao 266109, China; zqing1988@126.com; 6 College of Horticulture, Shenyang Agricultural University, Shenyang 110866, China; bcs@syau.edu.cn

Microbial metabolism exerts a pivotal effect on the ensiling process, a widely adopted technique for preserving forage crops as livestock feed. Silage manufacturing relies on the fermentation of plant substrates by lactic acid bacteria and other microbial taxa under anaerobic environments, with this fermentative process leading to a reduction in pH value, thereby suppressing the proliferation of spoilage microbes and maintaining the nutritional integrity of the silage. Furthermore, microbial interactions within the silage microecosystem modulate fermentation efficiency and its degree, hygienic properties, as well as livestock performance after ingestion. Gaining insight into the dynamics of microbial populations and communities in silage is indispensable for optimizing silage production, enhancing hygienic standards, and alleviating potential hazards linked to microbial contamination. This Special Issue, “Microorganisms in Silage”, comprises 11 original research articles and 1 communication and has received over 10,991 views.

Rape (*Brassica napus* subsp. *napus* L.) ranks as the world’s top oilseed crop, boasting extensive cultivation areas and a broad geographic distribution. Rape straw, the main by-product generated after seed collection, serves as a valuable biomass resource. In their study, Yang et al. (Contribution 1) explored how the combined application of *Lactiplantibacillus plantarum* and sucrose impacts the fermentation weight loss, silage quality, and microbial community composition of ensiled rape straw under different packing density regimes. Their findings indicated that enhancing packing density and adding the additives to rape straw silage could effectively mitigate fermentation weight loss, elevate silage quality, increase the relative abundance of beneficial lactic acid bacteria, and reduce the proliferation of undesirable microorganisms.

Selenium (Se) is an essential trace element for animals, exerting vital physiological functions such as participating in antioxidant reactions, regulating immune activity, and sustaining cardiovascular health. Numerous studies were conducted to promote the assimilation of inorganic Se into organic Se in plants, which can then be used as feed for livestock and poultry. This approach not only facilitates healthy animal production but also enables the generation of Se-enriched livestock and poultry products. Consequently, it is crucial to first clarify how different Se application rates affect plant agronomic traits, as well as their forage storage and processing properties. Hence, Chen et al. (Contribution 2) examined the influence of Se fertilizer application on the fermentation quality, chemical composition, and bacterial community of *Pennisetum americanum* × *Pennisetum purpureum* cv Minmu 7 (HPM7). Their results revealed that the use of Se fertilizer was able to increase the Se content in HPM7, with the optimal application concentration ranging from 0.50 to 2.00 mg/kg. This optimal dosage promotes the metabolism of soluble carbohydrates, thereby improving both the fermentation quality and microbial abundance of HPM7 silage.

High-moisture corn grain silage (HMCS) is prepared by directly threshing, crushing, compacting, and sealing corn kernels for fermentation when they reach physiological maturity, with a moisture content of 28% to 32%. This process promotes the proliferation of lactic acid bacteria, producing a feed suitable for cattle, sheep, swine, chickens, and other livestock and poultry. HMCS boasts numerous advantages, such as long shelf life, high nutritional value, and favorable feeding effects. Nevertheless, quality issues often occur during the feeding stage in dairy farms, where high-moisture corn tends to develop hot spots that facilitate mold growth, increasing the risk of mycotoxin contamination and thus exerting adverse impacts on farm profits. To address this issue, Bao et al. (Contribution 3) evaluated the effects of additives on the aerobic stability, fermentation characteristics, and chemical composition of HMCS. Their findings indicated that fermenting freshly harvested corn kernels into silage remarkably enhanced the digestibility of the feed. Furthermore, the addition of *Lentilactobacillus buchneri* (LB) not only significantly improved the overall quality of HMCS but also markedly enhanced its aerobic stability. Consequently, the application of LB as an additive is recommended for the practical production of HMCS.

*Cordyceps militaris*, a common medicinal fungus of the phylum Ascomycota, contains multiple active ingredients that are utilized in the production of medicine and health products. The expansion of its artificial cultivation has resulted in substantial amounts of solid medium residues and mycelial waste. Studies have demonstrated that traditional Chinese medicine processing residues retain numerous active substances, which can be repurposed as silage additives to improve fermentation quality and enhance feed value. To mitigate the environmental burden posed by *C. militaris* residues, Wei et al. (Contribution 4) investigated the feasibility of using these residues as silage additives. Their results showed that adding *C. militaris* residue alone or in combination with *Lactiplantibacillus plantarum* improved alfalfa silage quality by increasing lactic acid content and reducing ammonia nitrogen levels, thereby offering a novel approach for the utilization of such residues.

Alfalfa (*Medicago sativa* L.) is widely cultivated globally due to its high protein content and being rich in essential vitamins and minerals. However, alfalfa’s high natural moisture content poses challenges for successful silage production, leading to excessive dry matter (DM) loss, extensive proteolysis, and high butyric acid accumulation—reducing the DM intake of ruminants and increasing milk contamination risks. Ensiling high-moisture alfalfa with peanut vine not only prevents nutrient loss during alfalfa wilting but also realizes the high-value utilization of agricultural waste (peanut vine). Previous studies have explored the optimal mixing ratio of high-moisture alfalfa and peanut vine, yet the effect of additives on improving the nutritional and fermentation quality of this mixed silage has remained uninvestigated. Accordingly, Jia et al. (Contribution 5) evaluated the adaptability and interaction of *Lactiplantibacillus plantarum*, cellulase, and tannin—used alone or in combination—on the fermentation quality, chemical composition, and microbial communities of alfalfa–peanut vine mixed silage. The results showed that the combined application of *L. plantarum*, cellulase, and tannin is a promising strategy for preserving fresh alfalfa–peanut vine mixed silage, providing a reference for the reuse of such agricultural by-products.

Ensiling forage at low temperatures frequently results in inferior fermentation performance and nutrient depletion. Nevertheless, traditional commercial inoculants struggle to adapt to such harsh cold environments. To resolve this issue, it is essential to explore lactic acid bacteria strains that exhibit low-temperature tolerance and the ability to rapidly initiate fermentation. Liu et al. (Contribution 6) assessed the efficacy of a cold-tolerant *Pediococcus pentosaceus* OL77 strain on oat silage. Their findings indicated that OL77 can effectively enhance the fermentation quality and nutrient retention of oat silage under low-temperature conditions (below 10 °C), providing a practical inoculant solution for cold regions.

Forage scarcity in semi-arid areas calls for the screening of optimal sorghum cultivars to produce high-quality silage. In their work, Zhang et al. (Contribution 7) systematically assessed varietal variations in agronomic traits, nutritional value, fermentation performance, and bacterial community structure of whole-plant sorghum silage. Their results revealed that the grain-type cultivar JN3 is the most suitable for whole-plant sorghum silage production in semi-arid regions, owing to its optimal balance between yield and quality, highlighting the significance of comprehensive cultivar evaluation and implying the potential of targeted microbial inoculants to boost silage quality.

Agricultural wastes like sugar beet byproducts and corncobs encounter obstacles during storage and conversion into animal feed, such as high fiber content and low microbe-substrate interaction efficiency. Lin et al. (Contribution 8) assessed the impacts of supplementing *Lentilactobacillus buchneri* and cellulase on the fermentation quality, microbial community structure, and in vitro fermentation rate of air-dried mixed silage composed of sugar beet tops and corncobs. The results demonstrated that the synergistic effect of *Lentilactobacillus buchneri* and cellulase improves the fermentation quality and microbial community structure of the sugar beet top–corncob silage, thereby enhancing its in vitro fermentation properties and providing valuable insights for the recycling of such agricultural wastes.

Chinese herbal medicine (CHM) residues stand out as a promising and eco-sustainable type of silage additive, capable of regulating fermentation processes and improving nutrient retention. Yun et al. (Contribution 9) explored the impacts of two specific CHMs—*Astragalus membranaceus* L. (Astragali Radix, AR) and *Inula helenium* L. (Inulae Radix, IR)—on the fermentation characteristics, nutritional components, and bacterial community structure of barley silage. Herbal additives can remarkably upgrade the fermentation quality and nutritional worth of barley silage through the selective regulation of microbial communities. Both AR and IR effectively inhibited spoilage bacteria while enriching beneficial *Lactobacillus* strains, which in turn promoted acidification processes and mitigated protein degradation. These results highlight the considerable potential of Astragali Radix and Inulae Radix as natural, efficient, and sustainable microbial modulators for silage production. They provide a feasible approach to enhancing silage manufacturing, particularly in areas where alternatives to commercial inoculants are in demand.

*Cyperus esculentus* L., generally referred to as tiger nut, is an annual herb belonging to the Cyperaceae family. Wang et al. (Contribution 10) utilized lactic acid bacteria (LAB) strains isolated from *C. esculentus* that exhibited confirmed tolerance to assess their influence on silage quality after inoculation and identify effective strategies for boosting the application value of tiger nut in livestock farming. This research verified that the endogenous active substances in the stems and leaves of *C. esculentus* exert concentration-dependent inhibitory effects on *Leb. buchneri*, *Lpb. plantarum*, and *Lcb. rhamnosus*, with notable variations in tolerance levels among different strains. Silage trials indicated that inoculation with *Lcb. rhamnosus* remarkably enhanced silage fermentation performance. The application of endogenous dominant LAB derived from *C. esculentus* can mitigate such inhibitory effects, facilitate acid production, and improve silage quality—providing a scientific foundation for feed optimization and the targeted application of probiotics.

Grape branches and leaves are abundant in proteins and polyphenols, boasting beneficial properties that endow them with the potential to be used as high-quality feed components. However, their low soluble sugar content and high fiber level render them susceptible to slow fermentation or spoilage during the ensiling process, leading to substantial nutrient loss. In view of this, Li et al. (Contribution 11) evaluated the impacts of inoculating *Lactiplantibacillus plantarum* and cellulase on the silage quality of grape branches and leaves. Their findings revealed that the combined use of *L. plantarum* and cellulase effectively improves the silage quality of this raw material. This study realizes the conversion of low-value grape branches and leaves into high-quality feed, which provides technical support for the resource utilization of grape foliage and contributes to alleviating the local feed shortage.

Heterofermentative lactic acid bacteria (LAB_he_) can enhance the aerobic stability of silages. Given that high-dry-matter silages are particularly susceptible to aerobic deterioration, a key question arose as to whether an in vitro assay could facilitate the screening of LAB_he_ strains tolerant to such conditions—specifically, high osmolality. Consequently, Martens et al. (Contribution 12) investigated two aspects: first, whether the newly formulated culture medium was as appropriate for LAB_he_ strains as it had been proven for homofermentative lactic acid bacteria (LAB_ho_) and second, how LAB_he_ performed in situ under high forage dry matter (DM) conditions (≥40% DM) in comparison with medium DM levels. The pH reduction, both in vitro and in situ, served as the indicator of LAB activity and, by extension, their viability. The findings revealed that the in vitro assay enables the qualitative evaluation of LAB_he_ osmotolerance, yet fails to provide a quantitative ranking among different LAB_he_ strains.

Collectively, based on the aforementioned analysis of the roles and dynamics of microorganisms in silage, the research and application of silage-associated microorganisms are set to face several prominent challenges in the future.

**1.**  **Deciphering Complex Microbial Interaction Mechanisms**

The silage microecosystem harbors a diverse array of microbial taxa, including lactic acid bacteria, spoilage microorganisms, and functional symbionts. However, the current understanding of microbial interactions within this system remains inadequate—most studies have focused on single strains or simplified communities, whereas the complex synergistic, competitive, and antagonistic relationships among multiple microbial taxa, as well as their regulatory pathways under different forage types and ensiling conditions, have not been fully elucidated. Deciphering these intricate interaction networks constitutes a core challenge for optimizing silage fermentation processes.

**2.**  **Enhancing Microbial Agent Stability and Adaptability**

Microbial inoculants (e.g., lactic acid bacteria preparations) are extensively employed to improve silage quality, yet their practical application is constrained by poor stability and environmental adaptability. Extreme temperatures, humidity fluctuations, and the complex indigenous microbial community in raw forages can suppress the colonization and metabolic activity of exogenous microbial inoculants. Developing microbial inoculants with robust stress resistance, high colonization capacity, and broad adaptability to diverse forage varieties and regional climatic conditions represents a key challenge for their industrialization.

**3.**  **Mitigating Risks of Pathogenic and Toxigenic Microbes**

The anaerobic environment of silage may promote the proliferation of pathogenic microorganisms (e.g., *Clostridium* spp.) and toxigenic fungi (e.g., *Aspergillus* spp.), which produce harmful toxins that pose threats to livestock health and food safety. Current control measures (e.g., pH regulation, additive application) have inherent limitations in achieving targeted inhibition of harmful microorganisms without compromising beneficial ones. Establishing efficient, green, and targeted control strategies to eliminate pathogenic and toxigenic risks while preserving the nutritional value of silage is thus a critical challenge.

**4.**  **Integrating Multi-Omics Technology for Microbial Dynamics Analysis**

Traditional microbial research methods are insufficient to comprehensively characterize the diversity, functional potential, and dynamic changes in microbial communities in silage. Although multi-omics technologies (e.g., metagenomics, transcriptomics, metabolomics) have been increasingly applied, challenges persist in data integration, functional validation of key genes, and correlation analysis between microbial metabolic profiles and silage quality. Realizing the systematic interpretation of silage microbial dynamics through integrated multi-omics approaches is a major research priority.

**5.**  **Balancing Fermentation Efficiency and Environmental Sustainability**

With the growing demand for green livestock production, silage fermentation processes need to strike a balance between fermentation efficiency and environmental sustainability. Many of the silage additives (e.g., chemical preservatives) currently employed may exert potential adverse effects on the environment, and it should also be noted that the large-scale production of microbial inoculants tends to consume substantial resources. Developing eco-friendly fermentation technologies (e.g., bio-based additives, waste-derived microbial resources) and optimizing ensiling processes to reduce carbon emissions and resource consumption therefore represents an urgent challenge.

